# Characterization of *Bacillus cereus* AFA01 Capable of Degrading Gluten and Celiac-Immunotoxic Peptides

**DOI:** 10.3390/foods10081725

**Published:** 2021-07-26

**Authors:** Jun Lu, Yong Wu, Juanli Yuan, Jin Yuan, Zhongliang Wang, Jinyan Gao, Hongbing Chen

**Affiliations:** 1 State Key Lab Food Science & Technology, Nanchang University, Nanchang 330047, China; lujun0010@outlook.com (J.L.); ericyo918@hotmail.com (Y.W.); chinese582@sina.com (J.Y.); yuan2jin@126.com (J.Y.); wangzhongl719@163.com (Z.W.); gaojy2013@ncu.edu.cn (J.G.); 2 Sino German Joint Research Institute, Nanchang University, Nanchang 330047, China; 3 School of Pharmacy, Nanchang University, Nanchang 330036, China; 4 College of Food Science & Technology, Nanchang University, Nanchang 214122, China

**Keywords:** gluten, immunotoxic peptide, degradation, *Bacillus cereus*, genome

## Abstract

Wheat gluten elicits a pro-inflammatory immune response in patients with celiac disease. The only effective therapy for this disease is a life-long gluten-free diet. Gluten detoxification using glutenases is an alternative approach. A key step is to identify useful glutenases or glutenase-producing organisms. This study investigated the gluten-degrading activity of three *Bacillus cereus* strains using gluten, gliadin, and highly immunotoxic 33- and 13-mer gliadin peptides. The strain AFA01 was grown on four culture media for obtaining the optimum gluten degradation. Complete genome sequencing was performed to predict genes of enzymes with potential glutenase activity. The results showed that the three *B. cereus* strains can hydrolyze gluten, immunotoxic peptides, and gliadin even at pH 2.0. AFA01 was the most effective strain in degrading the 33-mer peptide into fractions containing less than nine amino acid residues, the minimum peptide to induce celiac responses. Moreover, growth on starch casein broth promoted AFA01 to degrade immunotoxic peptides. PepP, PepX, and PepI may be responsible for the hydrolysis of immunotoxic peptides. On the basis of the potential of gluten degradation, AFA01 or its derived enzymes may be the best option for further research regarding the elimination of gluten toxicity.

## 1. Introduction

Celiac disease (CD) is an autoimmune enteropathy that occurs in genetically susceptible individuals who develop immune reactions to cereal gluten. Gluten is the major environmental factor responsible for CD development. Wheat gluten is a storage protein of the seed endosperm with two water-insoluble fractions, namely, alcohol-soluble gliadins and alcohol-insoluble glutenins. The bread-making performance can be related to the profile of gliadins and glutenins [1]. However, gluten is difficult to be completely digested by human digestive proteases, releasing Pro/Gln-rich peptides, such as the 13-, 19-, or 33-mer. After entry into the lamina propria, these peptides are deamidated by tissue transglutaminase and then presented by DQ2+ or DQ8+ antigen-presenting cells to CD4+ T cells. Once activated, the CD4+ T cells drive a T-helper-cell type 1 response that leads to intraepithelial and lamina propria infiltration of inflammatory cells, crypt hyperplasia, and villous atrophy. The clinical presentation of CD is variable, including intestinal and extraintestinal symptoms [2]. Epidemiological data show that CD affects the quality of life of about 1% of the population, and its incidence is increasing in adults and children of various ethnic groups worldwide [3].

The mainstay of treatment for CD is strict and life-long adherence to the gluten-free diet (GFD) [4]. However, the GFD is laborious and negatively affects the quality of life because of its expensive cost, limited variety, and poor sensory properties. Thus, better alternatives are necessary. One possibility is enzyme supplementation to destroy the T cell epitopes in gluten directly or to facilitate gastrointestinal proteolysis [5]. The prolyl endopeptidases from *Myxococcus xanthus*, *Sphyngomonas capsulata*, *Flavobacterium meningosepticum*, and *Aspergillus niger* and the glutamine endoproteases from germinating barley show appreciable degrading activities toward gluten [6]. The propyl endoprotease from *A. niger* (AN-PEP) could effectively digest gluten to nonimmunogenic fragments [7], but dietary components in the food matrix influence the efficiency of AN-PEP [8]. In addition, ALV003 (a mixture of glutamine endoprotease from germinating barley seeds and a prolyl endopeptidase from *S. capsulata*) can only degrade small quantities of gluten and cannot significantly improve histologic and symptom scores in 494 patients with CD versus a placebo [9]. Therefore, the discovery of novel, more effective glutenases may open new perspectives toward the elimination of gluten toxicity.

Recently, some gluten-degrading bacteria and gluten-degrading enzymes have been discovered and studied on the detoxification of gluten, such as *Fusarium graminearum* [10], *Microdochium majus* [11], *Bacillus* spp. [12], actinidin from kiwifruit [13], and a serine protease from *Burkholderia gladioli* [14]. *Bacillus cereus* can hydrolyze gluten and the 33-mer gliadin peptide [12], but some fragments that contain immunodominant T cell epitopes were released from the 33-mer peptide. In the present study, we found that *B. cereus* AFA01 could be effective in degrading the 13- and 33-mer peptides and destroying the T cell epitopes in the 33-mer peptide. In addition, the influence of *B. cereus* AFA01 grown on four different culture media on the degradation of immunotoxic peptides was investigated in detail. Furthermore, the whole genomes of three *B. cereus* strains were analyzed to explore the genes of enzymes that potentially degrade gluten and immunotoxic peptides.

## 2. Materials and Methods

### 2.1. Synthetic Peptides

The 33-mer gliadin peptide (LQLQPFPQPQLPYPQPQLPYPQPQLPYPQPQPF) was synthesized by GL Biochem Ltd. (Shanghai, China), and the 13-mer gliadin peptide (LGQQQPFPPQQPY) was synthesized by Sangon Biotech Ltd. (Shanghai, China). The purity (≥95%) and structure were tested by reversed-phase high-performance liquid chromatography (RP-HPLC) and liquid chromatography–electrospray ionization–mass spectrometry (LC–ESI–MS).

### 2.2. Strains and Culture Media

*Bacillus cereus* strains CH (Anyang Yuanshou^®^), 21155 (Beijing, China Center of Industrial Culture Collection), and AFA01 (CGMCC 21108, China General Microbiological Collection Center, Beijing, China) were stored in the State-Key Laboratory of Food Science and Technology in Nanchang University. Five culture media compositions were used in this study: Brucella agar (BA), gluten agar (GA) [15], Luria broth (LB) [16], MCG-1 [17], and starch casein broth (SCB) [18]. The compositions of these media are given in Table 1.

### 2.3. Degradation of Gluten

Glutenase activities were measured as described by Gutiérrez et al. [19] with modifications. Briefly, the three *B. cereus* strains were diluted to an OD_620_ = 1.0 after 48 h of incubation. Then, 10 µL aliquots were inoculated on GA plates and incubated at 37 °C for 24 h. The plates were observed for a clear zone around the bacterial colony and evaluated by measuring the diameter of the halo formed.

### 2.4. Degradation of Mixed Gliadins in Solution

A mixture of gliadins obtained from Sigma (St Louis, MO, USA) was used to test the gliadinase activities as described by Fernandez et al. [20]. Briefly, the gliadin stock solution was added to the cell suspension (OD_620_ = 1.2) to reach a final gliadin concentration of 250 μg/mL. After incubation for 0, 0.5, 1, 2, 4, and 8 h at 37 °C, 100 µL aliquots were boiled and dried using the RapidVap Evaporation Systems (Labconco, Fort Scott, KS, USA). Then, the pellets were analyzed on 10% SDS-PAGE as described by Laemmli [21]. Gels were stained with Coomassie Brilliant Blue R-250. The intensity of 34–43 kDa protein was calculated per lane by using Quantity One software (Bio-Rad, Hercules, CA, USA).

### 2.5. Degradation of Gliadin in Gel (Gliadin Zymography)

Gliadin degradation in gel was tested using a zymogram gel (8%) containing mixed gliadins (2 mg/mL; Sigma, St. Louis, MO, USA) as described by Gutiérrez et al. [19]. Briefly, cells contained in a 150 µL aliquot were used for electrophoresis (100 V at 4 °C). Gels were processed in renaturing and developing buffers at pH 2, 4, 7, and 9. After developing for 48 h at 37 °C, the gels were stained with 0.1% (*w/v*) Coomassie Brilliant Blue R-250 in 40% (*v/v*) methanol and 10% (*v/v*) acetic acid, followed by destaining.

### 2.6. Degradation of Immunotoxic Peptides

Hydrolysis of the 33-mer and 13-mer gliadin peptide fractions by *B. cereus* was tested as described by Caminero et al. [17] with modifications. The cell suspension density used was OD_620_ = 1.2, and the initial concentration of the peptides was 0.5 mg/mL for 33-mer and 0.25 mg/mL for 13-mer. After incubation for 0, 0.5, 1, and 2 h, 1 mL sample aliquots were boiled and filtered through a 0.22 mm MCE membrane and then analyzed using RP-HPLC and matrix-assisted laser desorption ionization–time-of-flight (MALDI–TOF).

### 2.7. Growth and Gluten Degradation

*Bacillus cereus* AFA01 was inoculated on BA, MCG-1, LB, and SCB and then incubated at 37 °C for 48 h. Afterwards, the cells from different culture media were incubated with the 33-mer and 13-mer gliadin peptides. The degradation test of peptides was performed as above. The initial concentrations of the 33-mer peptides were 0.5 and 1.0 mg/mL.

### 2.8. Complete Genome Sequencing of B. cereus

The genomic DNA of the three *B. cereus* strains was extracted using the Wizard^®^ Genomic DNA Purification Kit (Promega, Madison, WI, USA) in accordance with the manufacturer’s protocol. Purified genomic DNA was quantified with a TBS-380 fluorometer (Turner BioSystems Inc., Sunnyvale, CA, USA). High-quality DNA (OD_260/280_ = 1.8–2.0, >20 µg) was used to construct an ~10 kb library and then sequenced. The genome was sequenced by using a combination of Illumina sequencing platform and PacBio RS II Single Molecule Real Time platforms at Majorbio Bio-Pharm Technology Co., Ltd. (Shanghai, China). The complete genome sequence was assembled using the PacBio and Illumina reads. Each set of query proteins was aligned with the databases, and annotations of best-matched subjects (e-value < 10^−5)^ were obtained for gene annotation as described by Delcher et al. [22].

### 2.9. RP-HPLC

Aliquots of 10 µL samples were analyzed by a LC-20AT model system (Shimadzu, Kyoto, Japan), and a C18 column (4.6 mm i.d. × 250 mm, 5 mm, Inertsil WP300; GL Sciences, Kyoto, Japan) was used. The eluents used were as follows: (A) 0.1% trifluoroacetic acid (TFA) in water and (B) 0.1% TFA in acetonitrile. For analyzing the 33-mer gliadin peptide, a 30 min gradient of 25–50% buffer B was used. The flow rate was 0.8 mL/min, and the column temperature was 25 °C. For analyzing 13-mer gliadin peptide, a 20 min gradient of 14–34% buffer B was used. The flow rate was 1.0 mL/min, and the column temperature was 50 °C.

### 2.10. Mass Spectrometry

Degradation of the 33-mer and 13-mer gliadin peptides by *B. cereus* was tested by a XIMA Performance (Shimadzu, Kyoto, Japan) MALDI–TOF as described previously [23] with modifications. MALDI–TOF used was a XIMA Performance (Shimadzu, Kyoto, Japan). Peptide solutions were mixed with 1 μL of matrix solution on a 384-well target plate. Measurements were performed in reflection mode with an acquisition mass range of 500−5000 Da. Peptide Calibration Standard from Shimadzu was used to calibrate the data of the 33-mer peptide and its modified forms.

### 2.11. Statistical Analysis

Data were analyzed using ORIGIN 8.0 and SPSS for Windows (version 15.0; SPSS Inc., Chicago, IL, USA) following a one-way linear ANOVA model. Duncan’s multiple range test was applied for mean separation for significant differences among treatments at *p* < 0.05 significance level. The data are presented as mean ± standard deviation (S.D.).

## 3. Results

### 3.1. Degradation of Gluten and Gliadin by B. cereus

**Figure 1:**
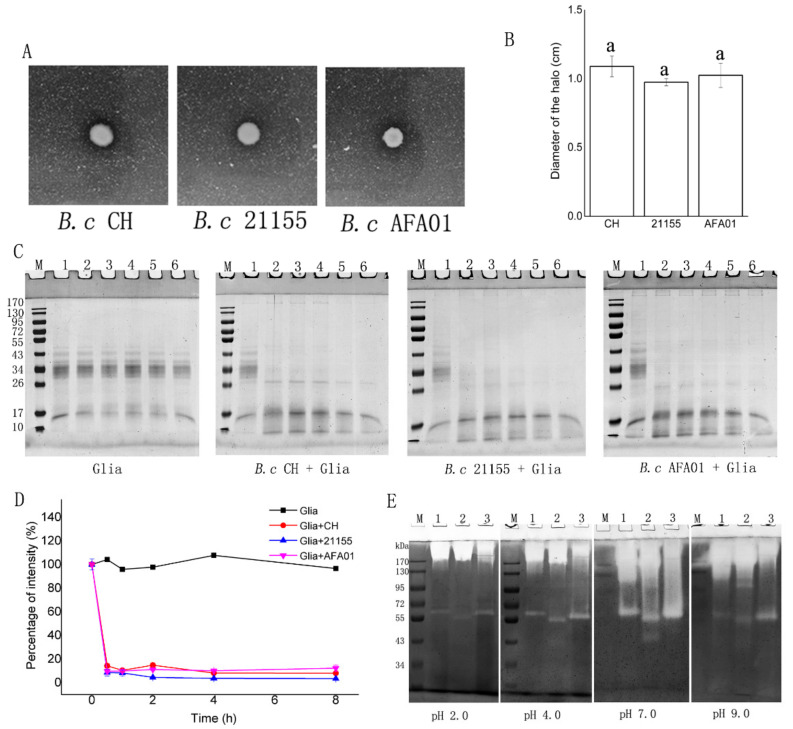
Glutenase activity of the three *B. cereus* strains. (**A**) Proteolytic zones in gluten agar plates that were inoculated with *B. cereus.* (**B**) The diameter of the halo formed in the gluten agar plates. The statistics are presented by the labels of ‘a’, which indicate non-significant (*p* > 0.05) differences. Columns represent the means ± SD. (**C**) The SDS-PAGE patterns of gliadin degradation in solution by the three *B. cereus* strains. Cells suspended to an OD_620_ 1.2. Mixed gliadins were added to a final gliadin concentration of 250 μg/mL. After t = 0, 0.5, 1, 2, 4 and 8 h incubation at 37 °C, aliquots of 100 μL were removed, heat-inactivated, and analyzed by 10% SDS-PAGE. M: molecular weight marker, Lane 1–6: bacteria + gliadin mixtures incubated for t = 0, 0.5, 1, 2, 4 and 8 h, respectively. (**D**) The percentage of 34–43 kDa protein bands in the gels was calculated by using Quantity One software (Bio-Rad). The data are presented as the means ± SD. (**E**) Gliadin zymography of three *B. cereus* strains at pH 2, 4, 7 and 9: 150 µL aliquots of cells (OD_620_ 5.0) were applied per lane. M: molecular weight marker, Lane 1: *B. cereus* CH; lane 2: *B. cereus* 21155; lane 3: *B. cereus* AFA01.

The GA culture medium, gliadin, and gliadin zymography were used to assess the gluten degradation activity of the three *B. cereus* strains (Figure 1). First, the GA culture medium in which gluten is the main nitrogen source was used to test the degradation of gluten. After 24 h incubation, the three strains grew on the gluten agar, created a clear zone, and the diameters of the halos formed had no significant differences (*p* > 0.05) (Figure 1A,B), indicating that the strains could equally hydrolyze the gluten proteins. Second, the commercial mixture of gliadins containing a variety of α/β-, ω-, and γ-gliadins was incubated with the three strains, and aliquots were analyzed by 10% SDS-PAGE after incubation for 0, 0.5, 1, 2, 4, and 8 h (Figure 1B). Gliadins were stained poorly with Coomassie and appeared as major bands in the 34–43 kDa region. Other minor components, including traces of albumins, globulins and glutenins, may also be present but were likely low in content. This study focused on the protease sensitivity of the 34–43 kDa protein bands. After 0.5 h incubation with the three *B. cereus* strains, the 34–43 kDa protein had undergone substantial degradation and they were virtually undetectable (Figure 1B), and the intensity of 34–43 kDa protein decreased to nearly 10% (Figure 1C). Third, gliadin zymography was used to characterize the approximate molecular weight and the pH activity profiles of the gliadin-degrading enzymes in the three *B. cereus* strains at pH 2.0, 4.0, 7.0 and 9.0 (Figure 1C). The enzymes from the three *B. cereus* strains could hydrolyze gliadin in gel at all pH values. The enzymatic activity was the highest at pH 7.0, and a weak but distinct band existed at pH 2.0, indicating enzymatic activity. All the strains produced an active enzyme band in the high-molecular-weight region (>170 kDa), possibly representing dimeric forms of the low-molecular-weight enzymes. They also had an active enzyme band in the ~55–72 kDa region, but not in the same location. In addition, *B. cereus* 21155 produced an active enzyme band in the ~43 kDa region at pH 7.0.

### 3.2. Peptidasic Activity against Immunotoxic Peptides

**Figure 2:**
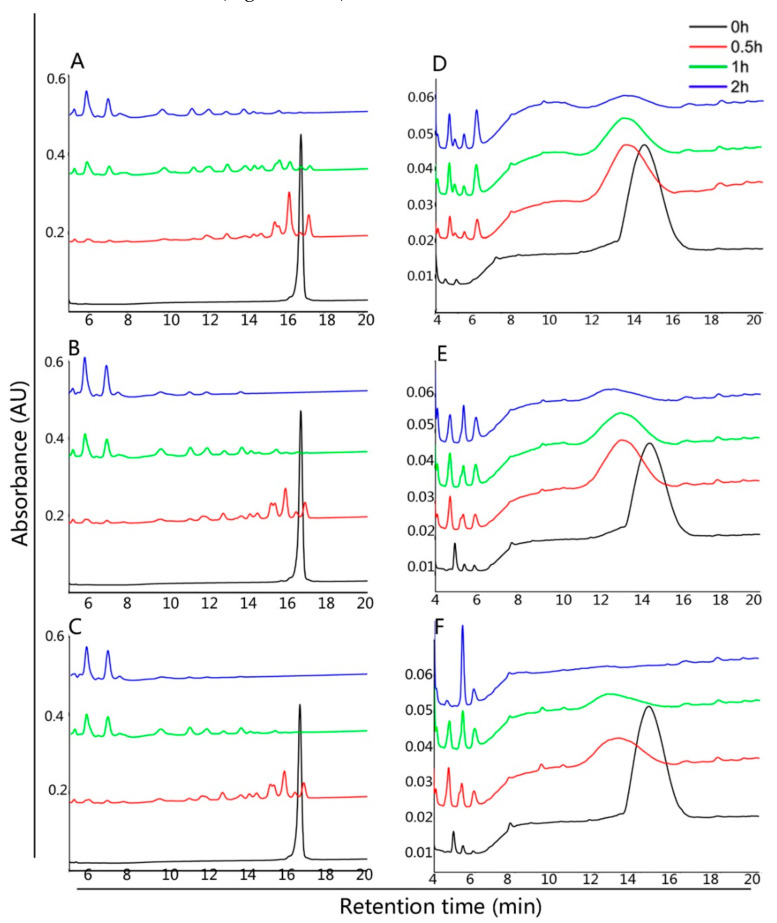
HPLC analysis of the degradation profiles of immunotoxic peptides. The cell densities used for the incubations were OD_620_ 1.2. The initial concentration of 33-mer gliadin peptide and 13-mer gliadin peptide was 0.5 mg/mL and 0.25 mg/mL. The 33-mer gliadin peptide was incubated with *B. cereus* CH (**A**), *B. cereus* 21155 (**B)** and *B. cereus* AFA01 (**C**) for 0, 0.5, 1 and 2 h, respectively. The 13-mer gliadin peptide was incubated with *B. cereus* CH (**D**), *B. cereus* 21155 (**E**) and *B. cereus* AFA01 (**F**) for 0, 0.5, 1 and 2 h, respectively.

**Figure 3:**
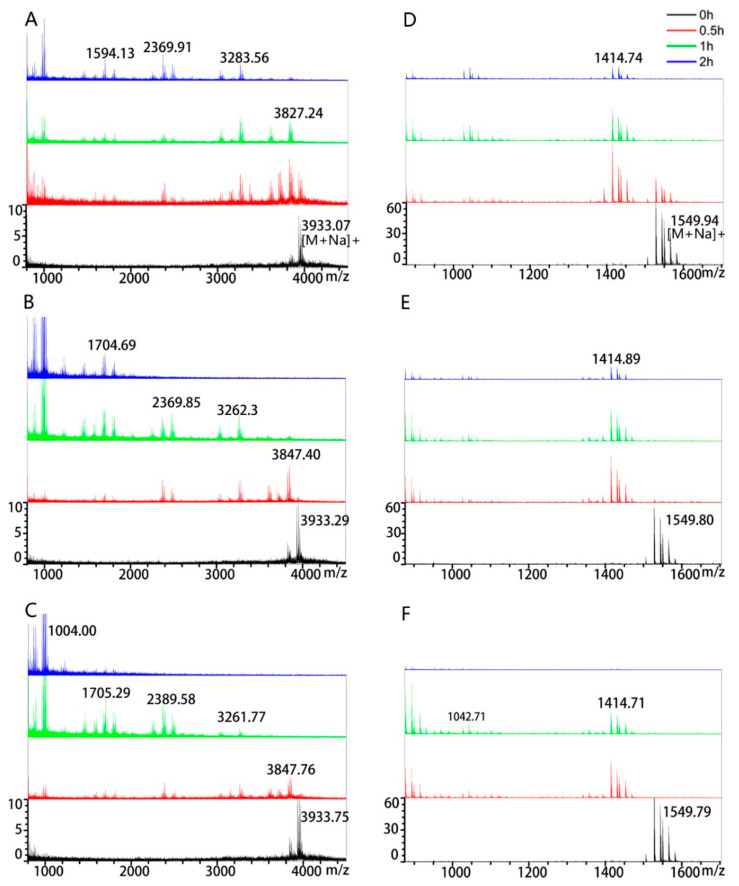
MALDI–TOF analysis of the degradation profiles of the immunotoxic peptides. The cell densities used for the incubations were OD_620_ 1.2. The initial concentration of 33-mer gliadin peptide and 13-mer gliadin peptide was 0.5 mg/mL and 0.25 mg/mL. The 13-mer gliadin peptide was incubated with *B. cereus* CH (**A**), *B. cereus* 21155 (**B**) and *B. cereus* AFA01 (**C**) for 0, 0.5, 1 and 2 h, respectively. The 13-mer gliadin peptide was incubated with *B. cereus* CH (**D**), *B. cereus* 21155 (**E**) and *B. cereus* AFA01 (**F**) for 0, 0.5, 1 and 2 h, respectively.

The proteolytic breakdown of the 33-mer and 13-mer gliadin peptides by *B. cereus* was tested by RP-HPLC (Figure 2) and MALDI–TOF (Figure 3), respectively. The intact 33-mer gliadin peptide eluted after 17 min, and the 13-mer gliadin peptide eluted after 15 min (Figure 2). In a suspension of bacteria, the 33-mer gliadin peptide completely degraded after 0.5 h of incubation, as evidenced by the disappearance of the peak at 17 min and the appearance of degradation fragments (Figure 2A–C). The 13-mer gliadin peptide was also cleaved by *B. cereus*, yielding fragments eluting between 5 and 8 min and between 12 min and 15 min (Figure 2D–F).

MALDI–TOF analysis showed that the molecular weights of the 33-mer and 13-mer gliadin peptides were ~3910 Da and ~1527 Da, respectively (Figure 3). After incubation with the three *B. cereus* strains, the intact peptides were degraded and some fragments appeared. After 2 h incubation with the 33-mer gliadin peptide, the residual peptides were no more than 1004 Da in *B. cereus* AFA01, whereas the residual peptides were more than 1594 Da in other strains (Figure 3A–C). The 13-mer gliadin peptide could also be degraded by the three *B. cereus* strains (Figure 3D–F). Some 1414 Da peptide residues were found in *B. cereus* CH and *B. cereus* 21155 after 2 h incubation, but not in *B. cereus* AFA01, which demonstrated the highest peptidase activity against the 33-mer and 13-mer gliadin peptides.

### 3.3. Influence of Media Composition on Protease Activity

**Figure 4:**
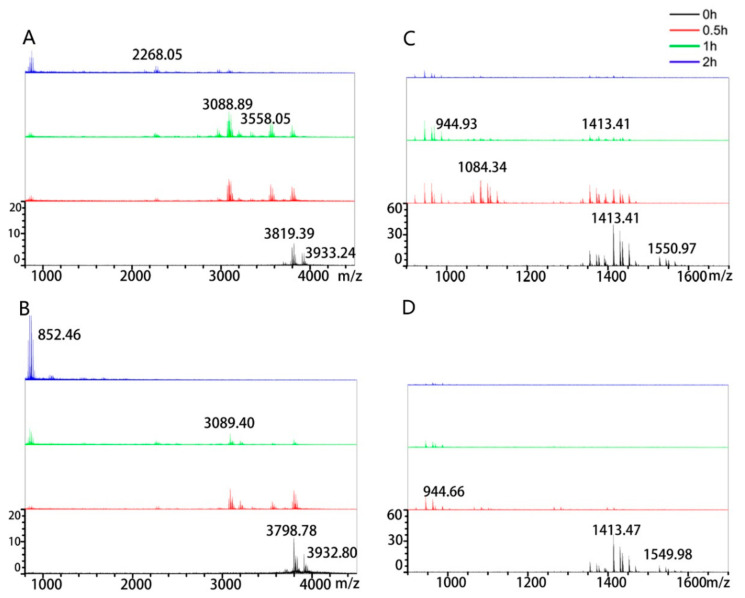
MALDI–TOF analysis of the degradation profiles of the 33-mer and 13-mer gliadin peptides. The 33-mer (1.0 mg/mL) gliadin peptide was incubated with *B. cereus* AFA01 from the medium of MCG-1 (**A**) or SCB (**B**) for 0, 0.5, 1 and 2 h. The 13-mer gliadin peptide (0.25 mg/mL) was incubated with *B. cereus* AFA01 from the medium of MCG-1 (**C**) or SCB (**D**) for 0, 0.5, 1 and 2 h, respectively.

The protease activity of microorganisms could be strongly influenced by culture parameters. Thus, the media components for achieving maximum enzyme activity were determined. The peptidase activity toward the 33-mer and 13-mer gliadin peptides by *B. cereus* AFA01 was tested in four types of media composition. AFA01 grown on BA medium cleaved 33-mer (0.5 mg/mL) into small peptides no more than 1004 Da after 2 h incubation. The 33-mer gliadin peptide was slightly hydrolyzed in the LB group, and 3798 Da peptide residues remained after 2 h. Notably, the residual peptides were no more than 874 Da after 1 h in MCG-1 and SCB groups (Supplementary Figure S1A,B). The 33-mer gliadin peptide (1.0 mg/mL) was incubated with AFA01 from the MCG-1 and SCB media to distinguish the effect of MCG-1 and SCB media on the degradation of the 33-mer gliadin peptide. The residual peptides were no more than 1076 Da after 2 h in the SCB group, except for the MCG-1 group (Figure 4A,B). A similar phenomenon occurred in the degradation of the 13-mer gliadin peptide (Supplementary Figure S1 and Figure 4C,D). The 13-mer gliadin peptide (0.25 mg/mL) was slightly hydrolyzed in the LB group, and 1415 Da residual peptides were found after 2 h. AFA01 cleaved the 13-mer gliadin peptide into small peptides no more than 944 Da after 2 h incubation in the BA group. The 13-mer gliadin peptide was partly degraded in the MCG-1 and SCB groups at 0 h. No peptides with MWs higher than 944 Da were detected after 1 h incubation with the strain from SCB medium. AFA01 grown on SCB medium more efficiently degraded the 33-mer and 13-mer gliadin peptides.

### 3.4. Whole Genome Sequencing and Bioinformatic Analysis of B. cereus

**Figure 5:**
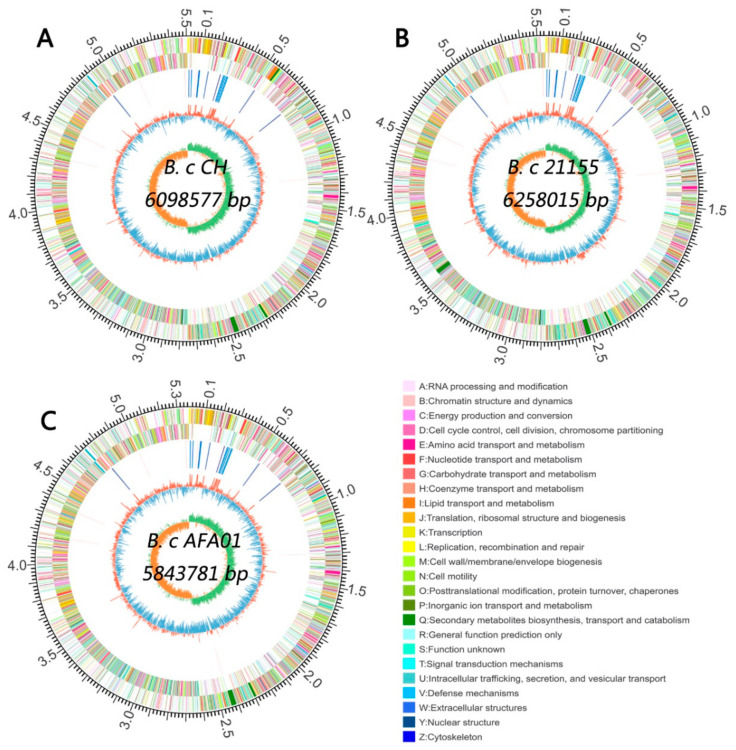
Genome map of *B. cereus* CH (**A**), *B. cereus* 21155 (**B**) and *B. cereus* AFA01 (**C**). Rings from the outside as follows: (1) scale marks (unit, Mb), (2) protein-coding genes on the forward strand colored by COG category, (3) protein-coding genes on the reverse strand (same color scheme as the second circle), (4) rRNA and tRNA genes, (5) GC content (deviation from average), and (6) GC skew in orange (below average) and green (above average).

Whole genome analysis was performed to decipher the complete set of genes involved in protein and peptide degradation. Whole genome assembly showed that the genome size, GC content, and genes with coding sequences were different in the three strains (Figure 5 and Table S1).

Analysis of enzymes acting on peptide bonds showed that the CH strain genome contained 99 genes, the 21,155 strain genome 94 genes, and the AFA01 strain 91 genes (Table 2). Among these genes, the number of genes encoding D-alanyl-D-alanine carboxypeptidase, zinc D-Ala-D-Ala carboxypeptidase, aminopeptidase S, microbial collagenase, bacillolysin, immune inhibitor A, sortase A, major intracellular serine protease, lantibiotic leader peptide-processing serine protease, repressor LexA, and proline iminopeptidase was different among the three strains. The number of genes encoding proline iminopeptidase was greater in AFA01 than in the other strains.

## 4. Discussion

Three *B. cereus* strains can efficiently hydrolyze gluten and gliadin fragments. These results were in agreement with the previous study that the *B. cereus* isolated from sourdough exhibits glutenase activity [12]. The GA culture medium in which gluten is the only nitrogen source could afford a reliable means of isolating microorganisms with glutenase activity. We used this medium to characterize the gluten degradation capacity of three *B. cereus* strains. As the size of the gliadin zymography clear zone was similar among the selected strains (Figure 1A), they were further evaluated for their gliadinase activity. Considering that the sequences in the α-gliadins contain immunodominant T cell epitopes, we focused on the protease activity toward α-gliadins. The molecular mass of α-gliadins is reportedly 28~35 kDa [24]. After 0.5 h incubation, the ≈34 kDa bands of gliadin substantially degraded (Figure 1B), confirming earlier gliadinase activity data of *Bacillus* species [25,26]. The three *B. cereus* (OD620 = 1.2) could degrade > 80% of gliadin (0.25 mg/mL) after 0.5 h incubation, while ALV003 [9] degraded an average of 80% of gluten (1 g) at a 300 mg dose in the same time. The smaller amount of *B. cereus* was more highly efficient in gluten degradation than ALV003. The three *B. cereus* strains could also degrade gliadin at pH 2.0 (Figure 1C). To our knowledge, only with *P. aeruginosa* activity, observed in recent studies, were enzymes mentioned to be active in gel at pH 2.0 [27], which was the lowest pH for gluten-degrading activity in the current work. The gluten molecular mass break-down pattern of active enzymes in our *B. cereus* strains (at the >170 kDa and ~43–72 kDa regions) (Figure 1C) was different from the data of other *B. cereus* strains (mentioning 28, 34, 35, and 58 kDa fragments) [28], suggesting that different enzymes may be active in the three *B. cereus* strains, which needs further purification and characterization.

We demonstrated experimentally that the three *B. cereus* strains can effectively cleave the 33-mer and 13-mer gliadin peptides. The intact 33-mer gliadin peptide (0.5 mg/mL) completely degraded after 0.5 h of incubation by the three *B. cereus* strains (Figure 2A), which was faster than the 2 h observed for other bacteria with 0.25 mg/mL 33-mer peptide [17,20,29]. The intact 33-mer gliadin peptide contains three overlapping immunodominant T cell epitopes, namely, PFPQPQLYP (one copy, MW ≈ 1085 Da), PYPQPQLPY (two copies, MW ≈ 1101 Da), and PQPQLPYPQ (three copies, MW ≈ 1066 Da) [30]. The residual peptides we found were smaller than the three immunodominant epitopes in the 33-mer gliadin peptide only from the *B. cereus* AFA01 group (Figure 3A). To detoxify, gluten peptide fragments should be less than nine amino acid residues [31]. Strain AFA01 also quickly degraded the 13-mer peptide into fragments of less than nine amino acid residues (Figure 3B). Together, the results indicated that *B. cereus* AFA01 was effective in detoxifying the 33-mer peptide and 13-mer peptides.

The rate of peptide degradation was different for the strains cultivated on the different media (Figure 4 and Supplementary Figure S1), which may be due to the protease activity of the strains being influenced by media components [32]. The tryptone and yeast in LB culture medium may be easy to be utilized by *B. cereus* AFA01, and the cells did not produce extra enzymes to hydrolyze the proteins, which may be the reason for low activity against the immunotoxic peptides. *B. cereus* can hydrolyze casein [33] and gluten [12], so AFA01 utilized the casein peptone, casein, and gluten. The SCB culture medium promoted the degradation of the immunotoxic peptides, which is consistent with the fact that casein can promote protease activity [34].

Bioinformatic analysis of the genome showed that the types of proteases and peptidases encoded by the three bacteria were similar, whereas the genes encoding for enzymes responsible for gluten degradation remained unclear. The genome size, GC content and genes with coding sequences of the three *B. cereus* strains (Table S1) in the present study were similar to those of previously reported *B. cereus* strains [35]. Genomic information provides insights into the mechanisms of bacteria, and the genome analysis of *Chryseobacterium taeanense* sp. 2RA3 revealed genes encoding proteases of the S9 family. A prolyl oligopeptidase was identified, and it degraded gluten immunogenic peptides in beer [36]. Then, we analyzed the genes catalyzing the hydrolysis of the C–N bond in the three *B. cereus* strains, and the number of those genes was similar among the three strains. However, there were no genes encoding the prolyl oligopeptidase in three *B. cereus* strains. To our knowledge, the enzymes degrading gluten have not been identified yet for *B. cereus*, and we did not confirm the genes that encoded enzymes to hydrolyze gluten yet. The genes encoding Xaa-Pro aminopeptidase (PepP), X-Pro dipeptidyl-peptidase (PepX), and proline iminopeptidase (PepI) were found in all three strains, and PepP, PepX, and PepI of lactic acid bacteria are proline-specific peptidases and these peptidases can degrade gliadin immunotoxic peptides [37,38], so we inferred that the genes encoding PepP, PepX, and PepI of the three strains may be responsible for the hydrolysis of immunotoxic peptides. Besides, the number of genes encoding PepI was greater in the genome of *B. cereus* AFA01 (Table 2) than in the genomes of the other strains used in our study. This result may be related to the higher activity of immunotoxic peptide degradation in *B. cereus* AFA01 than in the other strains.

It should be noted that this study has evaluated the degradation of gluten, gliadin, and gliadin immunotoxic peptides only by physicochemical methods. However, our results lack assessment of the gluten detoxification by *B. cereus* AFA01 with immunological methods. Moreover, we analyzed the whole genomes of three *B. cereus* strains and inferred that the genes encoding PepP, PepX, and PepI of the three strains may be responsible for the hydrolysis of immunotoxic peptides. Our results lack experimental data to confirm this hypothesis. The next work should demonstrate whether the genes encoding PepP, PepX, and PepI were responsible for degrading immunotoxic peptides by gene knockout studies. Despite its limitation, this study clearly indicated that *B. cereus* AFA01 effectively degraded gluten and immunotoxic peptides, and exhibited a high potential to detoxify gluten.

## 5. Conclusions

*Bacillus cereus* strains CH, 21155, and AFA01 can effectively degrade gluten, gliadin, and immunotoxic peptides, and the protease genes that may participate in gluten degradation were similar among the three strains. PepP, PepX, and PepI may be responsible for the hydrolysis of immunotoxic peptides. Among these strains, AFA01 was highly efficient in gliadin degradation and destroyed the immunotoxic T cell epitopes in the gluten fragments. The SCB culture medium could promote AFA01 to degrade immunotoxic peptides. This strain exhibited a high potential to detoxify gluten, where the produced enzymes may be especially useful for gluten detoxification in foods or during digestion to enhance the quality of life of patients with CD.

## Figures and Tables

**Table 1:** The compositions of five culture media.

Medium	Composition
BA	Peptone 10 g·L^−1^, Casein peptone 10 g·L^−1^, Yeast extract 2.0 g·L^−1^, Glucose 1.0 g·L^−1^, Sodium chloride 5.0 g·L^−1^, Agar 13 g·L^−1^, pH 7.0 ± 0.2
GA	Wheat gluten 23 g·L^−1^, Sodium chloride 5.0 g·L^−1^, Glucose 1.0 g·L^−1^, Sodium succinate 0.5 g·L^−1^, Soluble starch 1.0 g·L^−1^, Sodium pyruvate 1.0 g·L^−1^, Soluble pyrophosphate 0.25 g·L^−1^, L-Arginine 1.0 g·L^−1^, L-Cysteine 0.5 g·L^−1^, Haemin0.01 g·L^−1^, Vitamin K 0.001 g·L^−1^, Sodium bicarbonate 0.4 g·L^−1^, Agar 12 g·L^−1^
LB	Tryptone 10 g·L^−1^, Yeast extract 5.0 g·L^−1^, Sodium chloride 10 g·L^−1^
MCG-1	Glucose 20 g·L^−1^, Gluten 30 g·L^−1^, CaCl_2_ 0.05 g·L^−1^, ZnSO_4_ 0.07 g·L^−1^, L-cysteine 0.05 g·L^−1^, Tween 80 0.1%, 60 mM Phosphate Buffer (pH 6.5), Agar 16 g·L^−1^
SCB	Starch 10 g·L^−1^, Casein 3.0 g·L^−1^, KNO_3_ 2.0 g·L^−1^, NaCl 2.0 g·L^−1^, K_2_HPO_4_ 2.0 g·L^−1^, MgSO_4_ 0.05 g·L^−1^, CaCl_2_ 0.02 g·L^−1^, FeSO_4_ 0.01 g·L^−1^, pH 7.2

**Table 2:** The number of genes encoding enzymes acting on peptide bonds.

		The Number of Genes		
KO ID	KO Description	CH	21155	AFA01
K01297	muramoyltetrapeptide carboxypeptidase [EC:3.4.17.13]	1	1	1
K07258	serine-type D-Ala-D-Ala carboxypeptidase [EC:3.4.16.4]	4	4	4
K01299	carboxypeptidase Taq [EC:3.4.17.19]	1	1	1
K08602	oligoendopeptidase F [EC:3.4.24.-]	3	3	3
K03798	cell division protease FtsH [EC:3.4.24.-]	1	1	1
K01265	methionyl aminopeptidase [EC:3.4.11.18]	3	3	3
K19689	aminopeptidase [EC:3.4.11.-]	3	3	3
K01258	tripeptide aminopeptidase [EC:3.4.11.4]	2	2	2
K01255	leucyl aminopeptidase [EC:3.4.11.1]	1	1	1
K03100, K12380	signal peptidase I [EC:3.4.21.89]	7	7	7
K03101	signal peptidase II [EC:3.4.23.36]	1	1	1
K08600	sortase B [EC:3.4.22.71]	1	1	1
K02236	leader peptidase (prepilin peptidase)/N-methyltransferase [EC:3.4.23.43 2.1.1.-]	1	1	1
K05995	dipeptidase E [EC:3.4.13.21]	1	1	1
K01270	dipeptidase D [EC:3.4.13.-]	1	1	1
K01273	membrane dipeptidase [EC:3.4.13.19]	1	1	1
K08651	thermitase [EC:3.4.21.66]	1	1	1
K17733	peptidoglycan LD-endopeptidase CwlK [EC:3.4.-.-]	1	1	1
K01419	ATP-dependent HslUV protease, peptidase subunit HslV [EC:3.4.25.2]	1	1	1
K01338 K04076	ATP-dependent Lon protease [EC:3.4.21.53]	1	1	1
K01358	ATP-dependent Clp protease, protease subunit [EC:3.4.21.92]	2	2	2
K20742	gamma-D-glutamyl-L-lysine dipeptidyl-peptidase [EC:3.4.14.13]	1	1	1
K01304	pyroglutamyl-peptidase [EC:3.4.19.3]	1	1	1
K08777	neutral peptidase B [EC:3.4.24.-]	1	1	1
K21472	peptidoglycan LD-endopeptidase LytH [EC:3.4.-.-]	1	1	1
K21471	peptidoglycan DL-endopeptidase CwlO [EC:3.4.-.-]	1	1	1
K11749	regulator of sigma E protease [EC:3.4.24.-]	1	1	1
K06383	stage II sporulation protein GA [EC:3.4.23.-]	1	1	1
K06402	stage IV sporulation protein FB [EC:3.4.24.-]	1	1	1
K06399	stage IV sporulation protein B [EC:3.4.21.116]	1	1	1
K06012	spore protease [EC:3.4.24.78]	1	1	1
K14647	minor extracellular serine protease Vpr [EC:3.4.21.-]	1	1	1
K03797	carboxyl-terminal processing protease [EC:3.4.21.102]	1	1	1
K08303	putative protease [EC:3.4.-.-]	2	2	2
K19701	aminopeptidase YwaD [EC:3.4.11.6 3.4.11.10]	1	1	1
K01271	Xaa-Pro dipeptidase [EC:3.4.13.9]	2	2	2
K01262	Xaa-Pro aminopeptidase [EC:3.4.11.9]	1	1	1
K01281	X-Pro dipeptidyl-peptidase [EC:3.4.14.11]	1	1	1
K01286	D-alanyl-D-alanine carboxypeptidase [EC:3.4.16.4]	12	12	10
K07260	zinc D-Ala-D-Ala carboxypeptidase [EC:3.4.17.14]	7	5	3
K19702	aminopeptidase S [EC:3.4.11.24]	1	0	0
K01387	microbial collagenase [EC:3.4.24.3]	5	4	5
K01400	bacillolysin [EC:3.4.24.28]	1	1	2
K09607	immune inhibitor A [EC:3.4.24.-]	4	3	4
K07284	sortase A [EC:3.4.22.70]	2	4	2
K13275	major intracellular serine protease [EC:3.4.21.-]	3	1	1
K20486	lantibiotic leader peptide-processing serine protease [EC:3.4.21.-]	2	0	0
K01356	repressor LexA [EC:3.4.21.88]	2	3	2
K01259	proline iminopeptidase [EC:3.4.11.5]	2	3	4
Total		99	94	91

## Data Availability

The Genome Accession of Bacillus cereus CH, B. cereus 21155, and B. cereus AFA01 are CP068717-CP068718 (https://www.ncbi.nlm.nih.gov/nuccore/?term=CP068717:CP068718[accn]), CP068719-CP068723 (https://www.ncbi.nlm.nih.gov/nuccore/?term=CP068719:CP068723[accn]), and CP068724-CP068728 (https://www.ncbi.nlm.nih.gov/nuccore/?term=CP068724:CP068728[accn]) in NCBI.

## Supplementary Materials

The following are available online at https://www.mdpi.com/article/10.3390/foods10081725/s1, Figure S1: MALDI–TOF analysis of the degradation profiles of the immunotoxic peptides, Table S1: Comparative genome statistics of *B. cereus*.
