# Supplementary material for: Characterization of Bacillus cereus AFA01 Capable of Degrading Gluten and Celiac-Immunotoxic Peptides
Source: Foods. 2021 Jul 26;10(8):1725. doi: 10.3390/foods10081725 (PMC8392533; doi:10.3390/foods10081725)
Supplement: Supplementary file 1 [file foods-10-01725-s001.zip › foods-1282592-supplementary.pdf]

Figure S1

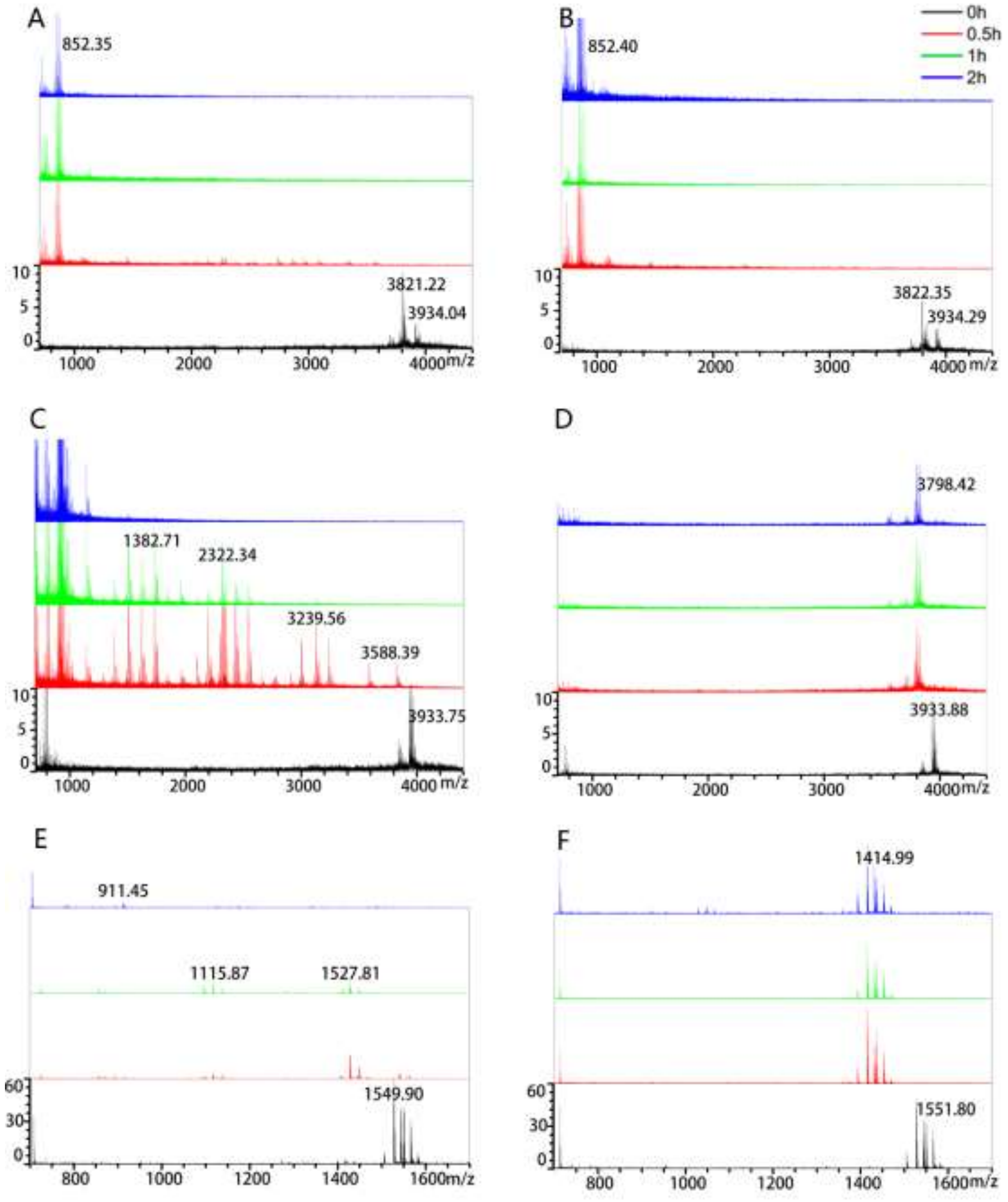

Figure S1. MALDI–TOF analysis of the degradation profiles of the immunotoxic peptides. The cell densities used for the incubations were OD_{620} 1.2. The 33-mer (0.5 mg/mL) gliadin peptide was incubated with *B. cereus* AFA01 from the medium of MCG-1 (A), SCB (B), BA (C) or LB (D) for 0, 0.5, 1 and 2 h. The 13-mer gliadin

peptide (0.25 mg/mL) was incubated with *B. cereus* AFA01 from the medium of BA (E) or LB (F) for 0, 0.5, 1 and 2 h, respectively.

Table S1 Comparative genome statistics of th *B. cereus*

	CH	21155	AFA01
**Genome Size (bp)**	6098577	6258015	5843781
**Chromosome No.**	1	1	1
**Plasmid No.**	1	4	4
**GC Content(%)**	34.92	34.91	35.22
**CDS No.**	6063	6362	5925
**tRNA No.**	106	107	107
**rRNA No.**	42	42	42
**Accession**	SAMN17369856	SAMN17369855	SAMN17369857
